# An equivalence evaluation of a nurse-moderated group-based internet support program for new mothers versus standard care: a pragmatic preference randomised controlled trial

**DOI:** 10.1186/1471-2431-14-119

**Published:** 2014-05-06

**Authors:** Alyssa CP Sawyer, John Lynch, Kerrie Bowering, Debra Jeffs, Jenny Clark, Christine Mpundu-Kaambwa, Michael G Sawyer

**Affiliations:** 1 School of Population Health, University of Adelaide, Adelaide, Australia; 2 School of Social and Community Medicine, University of Bristol, Bristol, UK; 3 Child and Family Health Service, Women’s and Children’s Health Network, Adelaide, Australia; 4 Discipline of Paediatrics, University of Adelaide, Adelaide, Australia; 5 Research and Evaluation Unit, Women’s and Children’s Health Network, Adelaide, Australia

**Keywords:** Nurse, Internet-based interventions, Early childhood, Mothers, Program evaluation

## Abstract

**Background:**

All mothers in South Australia are offered a clinic or home-visit by a Child and Family Health community nurse in the initial postnatal weeks. Subsequent support is available on request from staff in community clinics and from a telephone helpline. The aim of the present study is to compare equivalence of a single clinic-based appointment plus a nurse-moderated group-based internet intervention when infants were aged 0–6 months versus a single home-visit together with subsequent standard services (the latter support was available to mothers in both study groups).

**Methods/Design:**

The evaluation utilised a pragmatic preference randomised trial comparing the equivalence of outcomes for mothers and infants across the two study groups. Eligible mothers were those whose services were provided by nurses working in one of six community clinics in the metropolitan region of Adelaide. Mothers were excluded if they did not have internet access, required an interpreter, or their nurse clinician recommended that they not participate due to issues such as domestic violence or substance abuse. Randomisation was based on the service identification number sequentially assigned to infants when referred to the Child and Family Health Services from birthing units (this was done by administrative staff who had no involvement in recruiting mothers, delivering the intervention, or analyzing results for the study). Consistent with design and power calculations, 819 mothers were recruited to the trial. The primary outcomes for the trial are parents’ sense of competence and self-efficacy measured using standard self-report questionnaires. Secondary outcomes include the quality of mother-infant relationships, maternal social support, role satisfaction and maternal mental health, infant social-emotional and language development, and patterns of service utilisation. Maternal and infant outcomes will be evaluated using age-appropriate questionnaires when infants are aged <2 months (pre-intervention), 9, 15, and 21 months.

**Discussion:**

We know of no previous study that has evaluated an intervention that combines the capacity of nurse and internet-based services to improve outcomes for mothers and infants. The knowledge gained from this study will inform the design and conduct of community-based postnatal mother and child support programs.

**Trial registration:**

Australian New Zealand Clinical Trials Registry ACTRN12613000204741

## Background

During their initial postnatal weeks, in many parts of Australia, Canada, the United Kingdom, and other European countries, mothers and infants are supported by community nurses in family homes and community clinics [1,2]. This includes completing maternal and infant health checks, promoting parent knowledge and attitudes relevant to child rearing, and referring infants and mothers requiring additional help to appropriate specialist services.

In the past, nurse-based community services were the principal source of professional information and support for mothers of young children. However, during the last decade the internet has transformed the provision of healthcare services. For mothers of young children, the internet now provides free, convenient, and private access to health information [3,4], the opportunity to share and exchange information [5], and interactive treatment programs designed to address problems such as depression or anxiety [6]. Evidence about the extent to which the internet is consulted by mothers of young children is available from several recent studies [7-10]. For example, a study of 360 mothers of young children attending the Emergency Department at the Royal Children's Hospital in Melbourne found that 81% of mothers had access to the internet either at home or work and 43% had sought information from the internet about their children's health [3]. Wainstein and colleagues [7] reported that 83% of mothers attending a children's hospital responded that the internet had influenced the questions that they asked their doctor, and 18% reported that information obtained on the internet led to changes in their management of their children. As well, in our recent study of nurse home-visiting in South Australia (SA), preliminary analyses identified that in this population of mothers experiencing high levels of social adversity, 80% have access to the internet either at home, through a public library or via telephone [11].

The internet has the potential to reduce barriers to accessing services, including limited availability of skilled professionals, geographic isolation, the cost and inconvenience of travel and child care, and limited flexibility in work schedules [12]. The steadily increasing penetration of home computer and internet usage with vulnerable populations now renders delivery of intervention services via the internet a potentially valuable way to address the service needs of a high proportion of mothers and infants [12]. Potential cost reductions associated with transferring in-home programs to combined nurse-internet programs include reducing the need for service providers to physically travel to distant areas on a regular basis, avoiding costs of “no-show” visits, and allowing one professional to work with multiple families during a single day.

The frequency with which mothers seek online health information has encouraged the development of a large number of new websites and “phone apps”. However, an ongoing concern for professionals and mothers is the variable quality of information provided by these online sources of information [3,4,13,14]. For example, Plantin and Daneback [14] have reported that health-related information on the internet can be misleading and occasionally, “utterly wrong” [14-16]. There is also an almost total absence of evaluations assessing the ability of websites and “phone apps” to improve maternal and child outcomes [17]. We are not aware of any previous study that has evaluated the effectiveness of a nurse-moderated group-based internet support program employed to enhance post-natal outcomes for mothers and infants. The present study was designed to address these omissions.

### Objectives

The aim of the present study is to compare the effectiveness of a single clinic-based appointment plus a nurse-led group-based internet intervention when infants were aged 0–6 months versus a single clinic/home-visit plus standard services as requested by mothers (the latter was available to mothers in both study groups). Currently, the recruitment stage of this trial was completed in December 2013; intervention delivery and follow up assessments are ongoing.

Combining a clinic-based face-to-face mother/infant assessment with a 6-month nurse-moderated, group-based internet support program has several important benefits for the provision of services to mothers and infants. First, an internet-based program does not require the expensive provision of home visits by nurses. Second, it facilitates access to support for time-poor new mothers. Third, it provides nurses with online tracking of the extent to which mothers engage with different components of an intervention such as their participation in group discussions and utilisation of information available on a website. This allows nurses to more accurately target services to individual mothers and infants. Finally, it provides mothers with credible and readily accessible information about parenting and infant development.

## Methods/Design

### Study design

The evaluation is utilising a pragmatic preference randomised trial to compare the equivalence of outcomes for mothers and infants across the two study groups. We are examining the equivalence or “non-inferiority” [18] of the intervention with standard service because: (i) there is no evidence for the effectiveness of the current home-visit plus access to subsequent standard services, and (ii) while we believe the enhanced intervention has the potential for greater benefits to mothers and infants, given a local policy commitment to some form of post-natal nurse-based health checks, the key initial question for service providers is, “Is there a potentially more cost effective method of delivering a universal contact service for all mothers and infants in South Australia?” This implies that any new approach must produce outcomes that are equivalent or not inferior to current standard services. While the study is powered for equivalence, once we have established non-inferiority, we also have the ability to examine whether the enhanced nurse-internet intervention is superior on maternal and child health outcomes.

Outcomes will be assessed 4 times from the time infants are aged <2 months (pre-intervention assessment) through to 21 months. Outcomes will include parenting competence and self-efficacy (primary outcomes), maternal-infant attachment, maternal social support, role satisfaction and mental health, infant social and emotional development, and patterns of service use by mothers and infants (secondary outcomes).

The need for randomized controlled trials that are embedded in service practice and examine questions relevant to service clients, workforces, and delivery systems has been widely recognized in the medical and public health literature [19,20]. In contrast to explanatory trials which operate under ideal conditions, pragmatic trials occur within the context of current service delivery and population needs, and ask “Does this intervention work under usual conditions?” [19]

**Figure 1:**
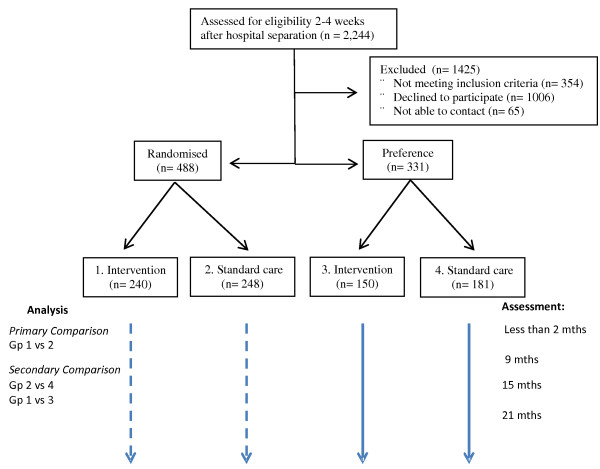
Participant flow diagram and overview of procedure.

We are using a preference-based design [21] in which service preferences are elicited from mothers at the time that they are recruited to the study. Mothers who express a “strong preference” for the intervention or for standard care are allocated to their preferred group. Mothers without strong preferences are randomized to intervention or standard care (Figure 1). The advantage of this approach is that many people refuse randomization, and/or drop out post randomization (if they don’t get their preferred service). As such, results can only be generalized to those who participate in randomization and complete the study (as low as 35% in some studies [21]). We have included those with a strong preference in this study as an “observational” cohort so we can compare outcomes in those randomized, with those who chose randomization or standard care, and thus improve generalizability of the findings to the whole population for whom the service is intended. If outcomes are similar in the randomized and preference groups then we can more clearly make inferences about the effects in the whole population.

### Setting

The Women’s and Children’s Health Network (WCHN) is the State-wide service responsible for “promoting, maintaining and restoring” the health of women, children and young people in South Australia [22]. Child and Family Health Services (CaFHS) is a key community component of the WCHN and is responsible for providing support for infants, children, and families across the State. To do this, CaFHS provides a full range of nurse home-visiting services, clinic-based nurse services, parent support groups, telephone support, and a specialist residential service for new mothers and infants with high needs.

### Procedure

Staff in birthing hospitals in South Australia ask all new mothers for their consent to be contacted by CaFHS to arrange a health check during their initial postnatal weeks. Approximately 93% of mothers give consent and are subsequently contacted by CaFHS administrative officers who arrange appointments for mothers with nurses either in family homes or at CaFHS clinics. For the purpose of the present study, mothers were advised by hospital staff about the study and the possibility that they may be asked to take part in it.

When subsequently contacted by CaFHS administrative officers, mothers were reminded about the study and verbal consent for their participation was sought. The script used by administration officers to inform mothers of the trial is included in Additional file 1. Mothers who consented were asked if they had a strong preference to participate in the internet-based group or receive standard care. As described above, those who expressed a strong preference for a particular arm of the study were enrolled in their preferred group. Those who did not have a strong preference were randomised to the intervention or standard care groups [23].

The contact details of mothers who gave verbal consent to participate in the study were provided to the research team, who contacted mothers by telephone and explained the study in more detail. Following the telephone call, mothers who confirmed their verbal consent were visited by a research assistant who completed a written consent process and arranged for completion of the 2-month (pre-intervention) assessment. At the time of their pre-intervention assessment, mothers were provided with a username and password for the internet-based intervention and given initial training in the use of the program. When they logged onto the program, mothers were welcomed to their group by the nurse group leader and proceeded with the intervention over the following six months.

### Participants

Participants eligible for the present study were mothers and infants who lived in regions where services are provided by one of six CaFHS clinics in Adelaide. These clinics were chosen because they provide services to a large number of mothers whose socio-demographic characteristics are comparable to the broader population of mothers in metropolitan Adelaide. Mothers were excluded from participation in the study if: (i) they did not have access to the internet, (ii) required an interpreter, or (iii) their nurse/clinician recommended that they not participate due to the presence of problems such as infant ill-health, domestic violence or substance abuse.

During the period of recruitment, 3367 maternal and infant health checks were completed by staff in these clinics. Of these mothers 1123 were not approached due to reasons outside the control of the research team (e.g., during periods of high competing demands, or staff shortages). As a result, during the period of recruitment 2244 were invited to participate and assessed for eligibility for the trial (See Figure 1). Of mothers assessed for eligibility, 354 were not eligible due to lack of internet access, insufficient English skills, or clinician exclusion. This left 1890 mothers of whom 65 subsequently could not be contacted by the research team. Among those who could be contacted (n = 1825), 819 agreed to participate (response rate = 45%).

### Sample size

Our primary outcomes focus on mothers’ sense of competence and self-efficacy in areas relevant to parenting and problem solving with infants. These outcomes were chosen in consultation with CaFHS nurses who identified one of their primary goals as helping ensure parents feel more competent to manage challenges associated with the care of infants.

Given that the primary question for the trial focuses on testing the equivalence of the nurse-moderated group-based internet program versus standard care, we estimated that with a sample size of 200 per randomized group, we would have 80% power at alpha = 0.05 to detect a 0.25 standard deviation difference (inferiority range) between the home-visit and nurse led internet groups in the primary outcome. Thus we would have 80% power to test the hypothesis that the internet-based intervention is no more than 0.25 of a standard deviation inferior when compared to standard care on the primary outcome measures. These estimates were based on data from our current 2 year follow-up of the Nurse Home Visiting program [11]. Based on this earlier study we allowed for an attrition of 20% over 2 years in the present study. To take this into account, we aimed to recruit 240 mothers to be randomised to each study group. Figure 1 shows the participant flows in the study leading to the final trial sample.

### Randomisation

Randomisation was based on the service identification number serially assigned to all infants when they are referred to CaFHS from their birthing hospital (assignment is done by central administrative CaFHS staff who had no involvement in recruitment of mothers, delivery of the intervention, or the analysis of results for the study). Mothers of infants with an odd service identification number were assigned to the intervention group. Mothers of infants with an even service identification number were assigned to the comparison group. We used this approach because CaFHS administration officers recruited mothers to the trial in the course of their normal work responsibilities, and because this trial was pragmatic by design we judged this method as being desirable because it demonstrated that randomisation could be done within normal workflows. However, this placed an additional burden on administrative staff in circumstances where they were already busy with a range of tasks. Despite this, they agreed to recruit mothers provided that the additional time demands were kept to manageable proportions. The use of a more traditional randomisation approach had the potential to increase recruitment time and interfere with administrative officers’ routine work. For this reason we chose to utilise the infant’s identification number, which was readily available to the administrative officers, to determine the group to which mothers with no strong preference were assigned.

The research team was blind to group allocation at the time of recruitment and assignment of mothers to the study groups. However due to the nature of the intervention, after the intervention commenced, it was not possible to keep research staff blind to the groups to which mothers had been allocated.

### Intervention and comparison condition

#### Standard care

As noted, in South Australia all families of newborns are offered a clinic or home-visit by a Child and Family Health community nurse during the initial postnatal weeks. The aim of the home-visit is to: (i) complete maternal and child health checks, (ii) provide comprehensive, information-based support to families of new infants, (iii) offer guidance and information about future child development, and (iv) link families to other services where this is required [24]. As this service is offered to all families of newborns in South Australia, it is relatively expensive involving more than 18,000 visits to homes across the State annually with each visit lasting about 60–90 mins. Although clinic visits are an option (or a visit in a ‘safe place’ for women who are considered not safe enough to visit at home) most parents prefer a home visit. Following this visit, mothers are encouraged to bring children for health checks at community clinics when the children are aged 6 and 18 months. A range of other services are also available at over 120 clinic sites across South Australia, with additional support including a telephone helpline, day-long support at community clinics for parents who need additional support with problems such as feeding or settling; and residential care for families with major unresolved problems with infant feeding, settling, and sleeping problems.

#### Nurse led group-based internet program

Following their first contact with the Service, all mothers in the intervention were assigned to an internet-based mothers’ group comprised of 12 mothers of similar-aged infants moderated by a trained Child and Family Health community nurse. The internet-based groups function in a comparable fashion to “chat rooms” found on many internet sites. However in the present intervention, all groups are nurse-moderated. Nurses utilise the group format to: (i) provide information directly to mothers, guided by a curriculum widely utilised in CaFHS face-to-face parenting groups, (ii) respond to questions asked by mothers, (iii) sensitively correct misperceptions and misinformation arising during discussion and exchange of information by mothers, and (iv) direct mothers to additional information sources both within the intervention website and via hyperlinks to other websites approved by CaFHS in SA.

The content of the intervention, established as a part of this project, addresses three broad issues: (i) steps that mothers can take to resolve common practical problems experienced by mothers of young children (e.g., feeding, sleeping, and “settling”), (ii) approaches that mothers can take to look after their own health and well-being, including problems with mood and depressive symptoms, and (iii) activities that mothers can use to promote the health of their infants (e.g., improving parent-infant attachment, stimulating infant language development). In the intervention mothers are guided to information relevant to infants at different stages of development within this period (e.g., 6 weeks, 4 months, and 6 months). We believe that this is important because clinical experience suggests that mothers want information specifically relevant to the age of their infant, rather than more broadly-based anticipatory advice about what might occur in the future.

The ‘mother’s view’ of the website is comprised of four components accessed by browser tabs: (i) Home Group - contains the chat room and also displays profile pictures (when supplied by participants) of other group members. Mothers’ and nurses’ posts and comments in the chat room are visible to all group members. The format of the chat room is similar to Facebook as this is familiar to many mothers, (ii) Milestones and Reminders - provides an interactive display of child developmental milestones and health reminders that can be printed locally. It also contains an interactive events calendar displaying topics that nurses will discuss, and other material relevant to the functioning of the group, (iii) Resources – contains ‘Frequently Asked Questions’ grouped into topic areas that parallel the topics in the curriculum used by nurses. Nurses can direct mothers to relevant resources as required or mothers can find information themselves, and (iv) Contacts and Assistance – contains a list of useful contact numbers and provides a portal through which mothers can privately message their group’s nurse.

The ‘nurses’ view’ of the website is comprised of three main elements: (i) Group Dashboard -which displays information about individual groups such as group activities, nursing notes maintained by nurses, and responses to quizzes posted by nurses to check maternal knowledge and to stimulate discussion between mothers, (ii) Parent Dashboard - which displays information about individual parents including parent case notes, individual website login activities (e.g., where parents view material but don’t post a message), and notifications that mothers have added information about children’s milestones, and (iii) Nurse Home Group page - through which nurses access their group’s chat room but also contains additional resources that nurses utilise (e.g., information inserted into the group chat room such as messages, reminders, and short quizzes).

### Measures

Maternal and infant outcomes in all groups are being evaluated using age-appropriate questionnaires completed when infants are aged <2 months (pre-intervention), 9, 15 and 21 months. Questionnaires are administered by trained research assistants in mother’s homes or at another convenient location chosen by mothers. The measures in the various domains below were selected based on their wide use, validity, reliability, and comparability with data collected in the Longitudinal Study of Australian Children (LSAC) [25].

#### Primary outcomes

##### Quality of maternal parenting

*Parenting Stress Index* (*PSI*): The PSI is a widely used questionnaire designed to assess parent and child characteristics relevant to “parent–child systems” [26]. Items consist of statements with a five-point response scale with endpoints labelled ‘Strongly Agree’ and ‘Strongly Disagree’. Relevant scales assess maternal perceptions of parenting competence, the quality of parent–child relationships, and the impact of parenting responsibilities on autonomy and self-identity. We utilise the five scales from the PSI labelled Competence, Isolation, Attachment, Role Restriction, and Spouse. Each of these scales assesses an aspect of parenting which is an important goal for CaFHS services.

*LSAC Parenting Assessment Measures:* The questionnaires employed in LSAC are being utilised to assess parental warmth, parental irritability, and parental sense of self-efficacy [27]. Level of parental warmth is based on six items that assessed the frequency with which expressions of warmth, happiness or affection occurred in the mother-infant relationship. Mothers respond using a 5-point response scale on which the endpoints are labelled “never/almost never” to “always/almost always”. Level of irritability is based on five items that assess the frequency with which expressions of anger or irritability occurred in the mother-infant relationship. Mothers respond using a 10-point response scale on which the endpoints were labelled “not at all” to “all the time”. Finally, level of parental self-efficacy is based on four items that assess mothers’ perceptions of their ability to manage their child in different circumstances. Mothers respond using a 10-point response scale on which the endpoints were labelled “not at all how I feel” and “exactly how I feel”.

#### Secondary outcomes

##### Infant-mother attachment relationship

*Parenting Stress Index (PSI) Attachment Scale:* The Attachment scale of the PSI assesses the quality of the mother-infant attachment relationship.

#### Infant social and emotional development

*Ages and Stages Questionnaire - Social-Emotional (ASQ:SE):* The ASQ:SE is used to measure the social and emotional development of infants [28]. Questionnaire items address: self-regulation, compliance, communication, adaptive functioning, autonomy, affect, and interaction with people. The ASQ: SE is comprised of eight questionnaires containing items developmentally appropriate for children aged 6, 12, 18, 24, 30, 36, 48, and 60 months. Each questionnaire can be used within 3months for children aged 6 through 30 months and within 6months for children aged 36 through 60 months. The 6-, 12-, 18- and 24-month questionnaires are being utilised in the present study. The number of items comprising the questionnaires range from 19 items on the 6-month questionnaire to 26 items on the 24-month questionnaire. All the questionnaires use a 3-point response scale on which responses are labelled “most of the time”, “sometimes”, or “rarely or never”.

#### Infant communication development

*Communication and Symbolic Behaviour Scales Developmental Profile - Infant/Toddler Checklist (CBS-DP):* Infants’ communicative abilities and symbolic ability will be assessed using the 24-item CBS-DP [29]. The measure provides a total score which can range from 0–57, as well as composite scores for the domains of social, speech, and symbolic skills. These domains are broadly related to infants’ pre-linguistic abilities (e.g., emotion, use of eye gaze, and gestures), linguistic abilities (e.g., use of sounds and words), and cognitive abilities (e.g., understanding of words and use of objects). The instrument has sound psychometric properties and normative data are available from LSAC (Commonwealth of Australia, 2011).

#### Parents’ perceptions of the quality of nursing support

Parents’ perceptions about the quality of the support provided by nurses will be assessed using a questionnaire specifically developed for this purpose. This will enable comparison of parents’ perceptions of the quality of nurse support in the nurse led internet-based program versus home-based visit. The questionnaire is comprised of 18 items which ask about the level of helpfulness of the nurses, the quality of the parent-nurse relationship and the extent to which mothers understood the goals of the program with which they were involved [30,31].

#### Service utilisation

We are identifying utilisation of community and clinic-based services by infants and mothers by means of standard questionnaires employed in the LSAC [27] and the National Child and Adolescent Mental Health Survey [32]. These items identify: (i) services used by mothers for their child during the previous 12 months, (ii) whether there are other services that children needed but could not access, and (iii) reasons why their child is unable to access needed services.

#### Demographic information

Background information is obtained about participating infants and their careers, including children’s age and gender, parental education and employment, housing, financial strain, and family characteristics (e.g., single-parent or two-parent; and the number and age of dependent children living in the household).

### Analysis plan

Primary analyses will be by intention-to-treat. For interim analyses, outcomes measured at one point in time during follow-up will be compared using linear or log binomial regression. For longitudinal analysis we will use Generalized Estimating Equations (GEE) to fit random effects regression models to describe the effects of the intervention on outcomes. GEE models are a flexible structure that allows parameter estimation accounting for temporally correlated outcome data and design effects due to clustering by nurse, although as a proportion of total variance, clustering is often found to be small [33]. Comparison of the randomized versus observational groups will be conducted by pooling the whole study population and including dummy variables (and potentially interactions with time and baseline psychosocial adversity) indicating randomized versus preference-based participation in the nurse led internet-based program.

#### Ethics approval

Ethics approval was received from the WCHN Human Research Ethics Committee (approval number REC2368/4/14).

## Discussion

The broad goal of this randomised pragmatic preference trial was to develop and evaluate the effectiveness of a nurse-led group-based internet intervention that combines the skills of Child and Family Health community nurses and the capacity of the internet to provide enhanced support for mothers of infants and young children. The work is based on the premise that effective linkage of nurse-based and internet-based services has the potential to cost-effectively enhance outcomes at a population level for mothers and children.

The advantage of conducting the present study in the service setting where the intervention could be utilised in the future is the increased likelihood that, if proved effective, it would be implemented in practice and utilised by regular clinic staff. The strong partnership with senior nursing staff in CaFHS during the design of the intervention has also helped ensure that the content of the intervention has high relevance to nursing practice and service goals.

Improving early childhood outcomes has been recognized as a policy priority internationally and nationally [34,35]. Achieving this goal requires cost-effective interventions which improve early childhood health and well-being at a population level. In many countries, including Australia, population-level maternal and infant services are provided via relatively expensive universal nurse home-visiting programs. However, it is possible that for many mothers, services could be just as effectively provided by clinic-based nurses supported by internet-based programs. This would allow more cost-effective use of home-visits to support those mothers and infants who need more intensive support.

## Abbreviations

ASQ:SE: Ages and stages questionnaire - social-emotional; CaFHS: Child and family health services; CBS-DP: Communication and symbolic behaviour scales developmental profile - Infant/toddler checklist; GEE: Generalized estimating equations; LSAC: Longitudinal study of Australian children; PSI: Parenting stress index; SA: South Australia; WCHN: The Women’s and Children’s Health Network.

## Competing interests

Kerrie Bowering is the Director of Child and Family Health Services (CaFHS), and Debra Jeffs is the Nursing Director of CaFHS. The authors have no other conflicts of interest to disclose.

## Authors’ contributions

AS drafted the manuscript with all authors contributing to revisions. The study design, and components of the manuscript were first conceptualised by MS, JL, KB, and DJ. All authors have approved the final manuscript as submitted.

## Pre-publication history

The pre-publication history for this paper can be accessed here:

http://www.biomedcentral.com/1471-2431/14/119/prepub

## Supplementary Material

Additional file 1 Script used by CaFHS administration officers to inform mother’s about the trial whilst contacting mothers to book a first contact visit (i.e., Universal Contact Visit). Click here for file
