# Supplementary material for: An equivalence evaluation of a nurse-moderated group-based internet support program for new mothers versus standard care: a pragmatic preference randomised controlled trial
Source: BMC Pediatr. 2014 May 6;14:119. doi: 10.1186/1471-2431-14-119 (PMC4108010; doi:10.1186/1471-2431-14-119)
Supplement: Additional file 1 — Script used by CaFHS administration officers to inform mother’s about the trial whilst contacting mothers to book a first contact visit (i.e., Universal Contact Visit). [file 1471-2431-14-119-S1.doc]

**Additional file 1**

**Script used by CaFHS administration officers to inform mother’s about the trial whilst contacting mothers to book a first contact visit (i.e., Universal Contact Visit)**.

| *I am phoning to offer you an appointment for the universal contact service and to arrange a convenient time.*  *When the CaFHS nurse asked about contacting you she should also have given you an Information Sheet about a new study “New Technology for New Mums” which is a trial of a new Internet-based program for new mothers.*  *Firstly, do you have access to the Internet?* (If yes, continue.)  *Do you have an email address?* (If yes, continue.)  *Did you receive the Information sheet? Can you remember what it is about?* (If no, provide extra information below.)  The purpose of the new Internet-based program is to provide better support for mothers of babies aged 0-6 months. The program gives you access to accurate information about babies and their care, and access to CaFHS nurses. It also lets you join an online “Getting to Know Your Baby Group”. Each group has about 12 other mothers with babies that are about same age as yours. The group is supported and guided by a CaFHS nurse and provides mothers with a way to share information and practical ideas about parenting.  There are two groups of mothers in the study. Mothers in one group are offered their *universal contact service* at a CaFHS clinic, followed by access over a 6 month period to the new Internet-based support program. Mothers in the second group are offered an appointment in their home, followed by access to nurses at CaFHS clinics, but they don’t have access to the Internet-based program.  *If you would be happy to take part in the study I will book your universal contact visit accordingly. We would really appreciate your help but it is voluntary and you can withdraw at any time. Would you be happy to take part at this stage?*  (If no revert to usual CaFHS Procedure.) *That’s OK, thanks.*  (If yes, continue.) *Thanks, we appreciate your help with the study.*  *Everyone is being divided into two groups – one group has the universal contact service here in the clinic and then access to the Internet-program for 6 months; the other group has the universal contact service at home and then just the normal CaFHS services (without access to the Internet-program). I will randomly allocate you to a group unless you have a definite strong preference for one group or the other. Do you have a strong preference?*  (If yes, allocation to preference).  (If no, randomly allocate on the basis of service identification number.)  *OK, you will be in the: clinic-Internet-program group.*  OR*: home-visit group.*  *Thanks, someone from the research team will call you to explain the study further.*  (Revert to usual CaFHS Procedure for booking UCV) |
| --- |
